# An exploration of grip force regulation with a low-impedance myoelectric prosthesis featuring referred haptic feedback

**DOI:** 10.1186/s12984-015-0098-1

**Published:** 2015-11-25

**Authors:** Jeremy D. Brown, Andrew Paek, Mashaal Syed, Marcia K. O’Malley, Patricia A. Shewokis, Jose L. Contreras-Vidal, Alicia J. Davis, R. Brent Gillespie

**Affiliations:** Department of Mechanical Engineering, University of Michigan, Ann Arbor, MI USA; Mechanical Engineering and Applied Mechanics, University of Pennsylvania, Philadelphia, PA USA; Department of Electrical and Computer Engineering, University of Houston, Houston, TX USA; School of Biomedical Engineering, Science and Health Systems (BIOMED), Drexel University, Philadelphia, PA USA; Department of Mechanical Engineering, Rice University, Houston, TX USA; Nutrition Sciences Department, College of Nursing and Health Professions, Drexel University, Philadelphia, PA USA; Department of Surgery, Drexel University College of Medicine, Philadelphia, PA USA; UM Orthotics and Prosthetics Center, University of Michigan, Ann Arbor, MI USA

**Keywords:** Sensory substitution, Grasp and lift, Joint torque feedback, Vibrotactile feedback, Prosthetics, Amputees

## Abstract

**Background:**

Haptic display technologies are well suited to relay proprioceptive, force, and contact cues from a prosthetic terminal device back to the residual limb and thereby reduce reliance on visual feedback. The ease with which an amputee interprets these haptic cues, however, likely depends on whether their dynamic signal behavior corresponds to expected behaviors—behaviors consonant with a natural limb coupled to the environment. A highly geared motor in a terminal device along with the associated high back-drive impedance influences dynamic interactions with the environment, creating effects not encountered with a natural limb. Here we explore grasp and lift performance with a backdrivable (low backdrive impedance) terminal device placed under proportional myoelectric position control that features referred haptic feedback.

**Methods:**

We fabricated a back-drivable terminal device that could be used by amputees and non-amputees alike and drove aperture (or grip force, when a stiff object was in its grasp) in proportion to a myoelectric signal drawn from a single muscle site in the forearm. In randomly ordered trials, we assessed the performance of N=10 participants (7 non-amputee, 3 amputee) attempting to grasp and lift an object using the terminal device under three feedback conditions (no feedback, vibrotactile feedback, and joint torque feedback), and two object weights that were indiscernible by vision.

**Results:**

Both non-amputee and amputee participants scaled their grip force according to the object weight. Our results showed only minor differences in grip force, grip/load force coordination, and slip as a function of sensory feedback condition, though the grip force at the point of lift-off for the heavier object was significantly greater for amputee participants in the presence of joint torque feedback. An examination of grip/load force phase plots revealed that our amputee participants used larger safety margins and demonstrated less coordination than our non-amputee participants.

**Conclusions:**

Our results suggest that a backdrivable terminal device may hold advantages over non-backdrivable devices by allowing grip/load force coordination consistent with behaviors observed in the natural limb. Likewise, the inconclusive effect of referred haptic feedback on grasp and lift performance suggests the need for additional testing that includes adequate training for participants.

## Introduction

Given recent advances in actuator and sensor technology, upper-limb prosthesis development has seen an explosion in innovation, moving devices closer to the physiological form and function of the natural limbs they are intended to replace. However, an amputee’s ability to control the additional degrees of freedom and realize function in even the simplest of tasks lags far behind. In the intact limb, dexterous control relies on efferent neural pathways carrying user intent to the musculoskeletal system and afferent pathways carrying sensory signals from mechanoceptors and proprioceptors back to the central nervous system. The returning sensory information is used in part for feedback control, and also to develop and refine internal models of the limb and environment for use in feedforward control [1, 2].

For upper-limb amputees, all efferent and afferent neural pathways end abruptly at the most distal point of the residual limb. Prosthetic limbs can, however, provide an artificial conduit through which efferent and afferent signaling can be established to a terminal device. But dexterous motor function requires an interface that reliably determines user intent and provides easily interpreted sensory feedback. In commercial devices, user intent is determined either by harnessing motion in other parts of the body (as in body-powered prostheses) or transducing electromyographic (EMG) signals (as in myoelectric prostheses). Terminal device aperture and object grip forces are available to body-powered prosthesis users in the form of displacement and tension in the Bowden cable. Users of myoelectric prostheses generally have no haptic sensory access to aperture or grip force and so must use incidental audio or visual cues to estimate aperture and grip force. This shortcoming has motivated research into the use of haptic display on the residual limb to relay signals encoding terminal device aperture or grip force. Actuator technologies being evaluated for referred haptic display include vibrotactile [3–12], skin stretch [9, 13], pressure (pushing normal to the skin) [9, 14–16] and joint torque (a torque applied across a joint by an exoskeleton) [17]. However, the use of haptic sensory feedback in myoelectric prosthetics has not been commercialized to date, with only one exception [18].

### Mixed results with referred haptic feedback

Based on well established tenets in teleoperation and haptics, feeding back force from interactions taking place in a remote or virtual environment should improve manual performance and dexterity and decrease task completion times [19–21]. The inverse also holds: if non-amputee participants are denied sensory feedback through temporary anesthetization of their digits, they lose the ability to accurately modulate grip force according to the weight of an object. Rather than adopting an economical grasp, they grip tighter than necessary to prevent slip. Also, grip force control degrades to the point that even with a safety margin in place, a larger number of slips occur [22]. Similar findings pertain for persons with impaired sensory afferents [23, 24]. Based on these observations, the lack of sensory feedback made available from a myoelectric prosthesis would be expected to produce a similarly compromised coordination and regulation of grip force during grasp and lift tasks performed by amputees using myoelectric devices.

A number of studies have shown that providing haptic feedback from a prosthesis leads to performance improvements in certain tasks. For example, Meek et al. [25] showed that delivering a pressure cue to the residual limb in proportion to grip force enabled non-amputee participants to select and maintain a grip force that both minimized slips and minimized crushing of a brittle object. Panarese et al. [14] showed that grip force displayed to the toes contributed to improved grasp and lift performance. Rombokas et al. [6] demonstrated virtual force-motion task improvements for electromyography with vibrotactile feedback. In our own previous work we evaluated joint torque feedback displayed using a motorized exoskeleton spanning the elbow for its potential to provide haptic feedback similar in nature to that produced by the shoulder harness of a body-powered prosthesis (about the same joint that generates the control input) [17, 26]. In particular, we found that a torque applied at the elbow in proportion to electronically sensed grip force facilitates discriminating objects by stiffness [27].

In certain studies, however, performance differences were not clear between the sensory feedback and no-feedback conditions, or differences only became evident for certain populations or when uncertainty was introduced into the feedback loop. Saunders and Vijayakumar [28] found that the utility of vibrotactile feedback delivered to non-amputees wearing a mock prosthetic hand was only evident when timing uncertainty was introduced into the open/close control triggering of the terminal device. Chatterjee [3] found that vibrotactile feedback was effective only for experienced users of myoelectric prostheses, and only for certain target grip forces. Cipriani et al. [29] found that vibrotactile feedback did not aid performance in the presence of vision. Ninu et al. [30] found that direct force feedback was not essential for the control of grasping force. As Saunders and Vijayakumar argue, feedfoward control is often sufficient and lack of feedback does not necessarily lead to a deterioration in performance. Sensory feedback may be used to tune internal models used in feedforward control rather than to reduce errors in an on-line feedback control process.

### Effect of terminal device impedance

While aiming to improve function in upper limb prosthetics with the addition of sensory feedback, we are led to consider the role of the mechanics of the prosthesis in producing that feedback. Sensory feedback, whether generated by sensory organs in an intact limb or by electronic sensors in a prosthetic limb, carries information not about the object in isolation, but about the behavior of an object in interaction with the intact or prosthetic limb. If the mechanics of a prosthetic limb differ from the mechanics of an intact limb, then the sensory feedback will likewise differ. That is, sensory feedback informs the user about the coupled dynamics between the limb and environment. The driving point impedance of the terminal device (in particular, the dynamic relationship between aperture and grip force) determines how the device responds to the mechanical properties of the objects it encounters. For example, if a terminal device is not back-driveable (has a high impedance) by virtue of a highly geared motor, then even the smallest compliance in the device or object will result in a grip force that does not decrease even when the EMG command signal is nulled. Aperture motion cannot be driven by the object; only by the motor^1^. To reduce the grip force, a commercial prosthesis user must drive the motor in the reverse direction, usually by producing an EMG signal from an antagonist muscle. Grip force trajectories in which the peak grip force is held until driven in the reverse direction can be observed in published results from grasp and lift tasks performed by amputees using non-backdrivable terminal devices [14, 28].

The role of the mechanics of a prosthetic device relative to sensory feedback might be further considered in light of teleoperator technology. By design, the master and slave devices of a teleoperator have low impedance so that the teleoperator becomes “transparent” and the user is able to interact as directly as possible with the remote environment. Ideally, the coupled dynamics involve only the mechanics of the body and environment without the intervening dynamics of the teleoperator (the master and slave devices and teleoperation controller). Similar design principles hold in body-powered prostheses, where a low impedance terminal device and maximally “transparent” transmission comprising the harness and Bowden cable ensures that the feel of the distal environment is masked as little as possible.

To maximize the utility of sensory feedback, we fabricated a prototype terminal device with a low backdrive impedance, using only a modest mechanical advantage between motor and aperture. We supposed that a terminal device whose mechanics are as close as possible to the mechanics of an intact limb would produce behaviors (and associated sensory signals) that are most easily anticipated and interpreted by users. Using a grasp and lift experimental paradigm, we expect to see grip and load force traces that are more indicative of grasp and lift tasks performed with an intact limb (e.g. [31]) than those performed with commercial non-backdrivable prosthetic limbs [14, 28].

In this paper we present the results of an experiment aimed at exploring the utility of sensory feedback from a back-driveable prosthesis whose aperture was driven to track a myoelectric signal using proportional control. We derived the myoelectric signal using a single bipolar electrode at a muscle site on the forearm. Proportional control of terminal device aperture ensured that a direct mapping of myoelectric signals corresponded to position control during free motion, and corresponded to force control once a stiff object was contacted. To investigate the effect of referred sensory feedback, our device conditionally relayed grip force back to our participants through one of two haptic displays—vibrotactile display or joint torque display. When denying our participants the ability to monitor grip force, we expected grasp and lift performance to degrade as it does for individuals lacking the full suite of sensory afferents in their natural hand [32]. In particular, we hypothesized that users would overcompensate and produce a grip force well above the slip threshold. We also expected an increased number of slips. Restoring grip force sensation through haptic feedback was expected to improve task performance by diminishing the overcompensating behavior and reducing the amount of object slips.

We recruited participants from two populations: amputees who were regular users of commercial myoelectric prostheses and non-amputees who had no prior experience using any type of myoelectric control. Our custom motorized terminal device could be adapted for use by trans-radial amputees and non-amputees alike. It denied non-amputees the mechanoception and proprioception that come from the cutaneous receptors in the hand. Thus the device was intended to place non-amputee participants on a level playing field with amputees. We asked all participants to use the gripper to grasp and lift an instrumented object whose weight could be changed covertly.

## Methods

### Experimental setup

Our experimental apparatus consisted of a myoelectric sensor and conditioning circuit, a motorized gripper, a motorized elbow brace, a vibrotactile display, and an instrumented object. The myoelectric sensor consisted of a signal and ground disposable surface electrode (Vermed SilveRest Resting EKG Electrode, 1 3/8" Vinyl Tape) and a custom conditioning circuit that provided full-wave rectification, low-pass filtering with a 3.4 Hz cutoff frequency, and variable amplification of the raw EMG signal. The EMG signals were measured from muscles in the forearm. For non-amputee participants these were muscles on the volar aspect of the forearm in the region of the wrist flexor and extrinsic finger flexor muscles. For amputees, these were the muscles in the residual limb usually used for control of a myoelectric prosthesis, as identified by the prosthetist member of the research team. In addition to the adhesive backing on the electrodes, a compression sleeve was used to keep the electrodes from coming loose during the experiment.

**Fig. 1:**
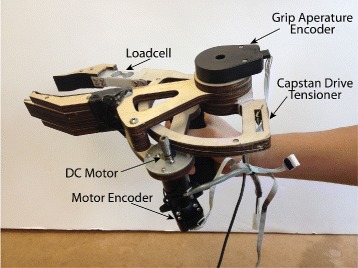
Motorized gripper. Gripper features motorized capstan drive and load cell

The motorized gripper was a custom designed prehensor driven by a motorized capstan drive featuring a Maxon RE 30 (60W) DC Motor (Fig. 1). The DC motor was powered by a 24V power supply (TDK-Lambda ZWS150PAF) and analog servo drive (Advanced Motion Control 12A8) that was tuned in current mode to have voltage to current gain of 1.0. The motor was equipped with a rotary encoder on the motor shaft (Maxon 1024 CPR) and a rotary encoder on the gripper axis (US Digital, 2500 CPR). In addition, the gripper was equipped with a 5 kg-capacity beam load cell (Transducer Techniques LSP-5). The gripper was capable of delivering 10N of force at the object contact surface. The gripper was mounted to the distal portion of the motorized elbow brace for amputee participants, and hand-held about a foam grip for non-amputee participants. This was done to keep the overall length of the apparatus (and any gravity moment it produced) similar between the two participant groups.

In operation the gripper was position-controlled from the EMG signals according to the following control law

(1) $$\begin{array}{*{20}l} G_{cmd}=K_{g} \cdot ({S}/{S_{max}}\cdot K_{a} - G_{apt}) + G_{b}  \end{array} $$

where *G*_*cmd*_ is the command to the gripper motor servo drive (in volts), *K*_*g*_ is the proportional gain constant, *S* is the EMG signal, *S*_*max*_ is the maximum possible EMG signal recorded during setup, *K*_*a*_ is a constant that converts the EMG signal to a desired aperture, *G*_*apt*_ is the actual gripper aperture, and *G*_*b*_ is an offset or bias. When the gripper was unconstrained, producing the maximum EMG signal *S*=*S*_*max*_ would completely close the gripper. When an object in the grasp of the gripper constrained its motion, commanding an aperture smaller than that allowed by the object would cause the error in Eq. 1 (*S*/*S*_*max*_·*K*_*a*_−*G*_*apt*_) to grow. This larger error resulted in a larger command signal *G*_*cmd*_ to the gripper motor, and therefore a larger torque produced by the motor. This torque produced a corresponding grip force on the object surface, and was measured by the load cell in the gripper.

**Fig. 2:**
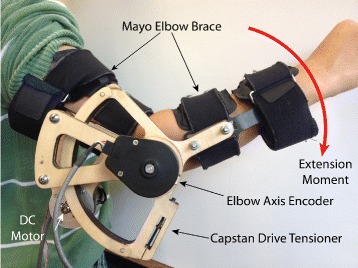
Motorized elbow brace. Right-handed elbow brace with motorized capstan drive. The brace produced an extension moment about the elbow proportional to force measured by the gripper

**Fig. 3:**
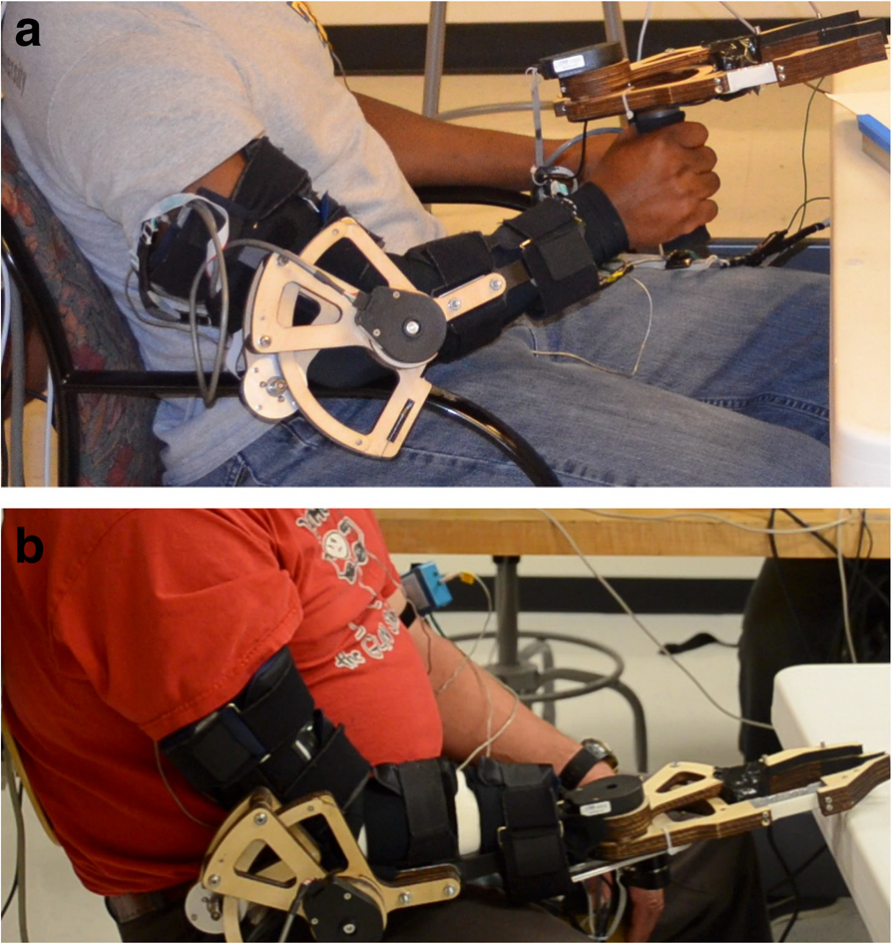
Testing setup. **a** Non-amputee participants hold the gripper in hand. **b** Amputee participants wear the gripper attached to the motorized elbow brace. In addition to the motorized elbow brace and gripper, participants are wearing the vibrotactile display

The motorized elbow brace was used to provide joint torque feedback in the form of an extension moment about the elbow joint. It consisted of a right-handed Aircast Mayo Clinic Elbow Brace customized with a capstan drive and electronics identical to that of the gripper (see Fig. 2). The motor was equipped with a rotary encoder on the motor shaft (Maxon 1024 CPR) and a rotary encoder on the brace shaft (US Digital, 2500 CPR). The motorized brace was capable of delivering 0.15N ·m of torque as an extension moment about the elbow. Participants’ arms were secured in the elbow brace through four velcro straps (see Fig. 3a). For amputee participants, custom cuffs were used in addition to the velcro straps (see Fig. 3b). The width of the brace could also be adjusted. In operation, the motorized brace produced an extension (or flexion) moment about the elbow proportional to the measured grip force using the formula

(2) $$\begin{array}{*{20}l} E_{cmd}=K_{e} \cdot F_{g},  \end{array} $$

where *E*_*cmd*_ is the command to the exoskeleton motor servo drive (in volts), *K*_*e*_ is the proportional gain constant, and *F*_*g*_ is the grip force measured by the gripper load cell.

**Fig. 4:**
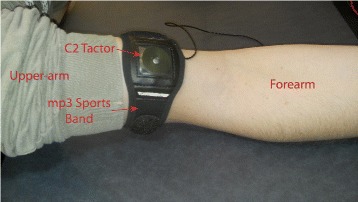
Vibrotactile display. C2 tactor inside mp3 sports band

The vibrotactile cue was adapted from Christiansen et al. [33], and was carefully designed considering human perceptual capabilities and prior psychophysical study results. The vibrotactile display (see Fig. 4) consisted of an Engineering Acoustics Inc. C2 tactor driven through an H-Bridge amplifier (LOGOSOL DC Servo Amplifier LS-5Y-12-DE). The tactor was held in place using an off-the-shelf mp3 player sports arm band. In operation, the vibrotactile cue was created by multiplying a sine wave of constant 250 Hz frequency by a sawtooth function of constant 10 Hz frequency. The amplitude of the cue *T*_*c*_ was exponentially proportional to the measured grip force and driven according to

(3) $$\begin{array}{*{20}l} T_{c}=0.5\cdot e^{2T_{c_{ref}}} \cdot T_{c_{amp}} \cdot \sin{\left(2\pi T_{c_{freq}}t\right)}  \end{array} $$

with $T_{c_{\textit {amp}}} = 10t-\lfloor 10t\rfloor $, $T_{c_{\textit {freq}}} = 250~Hz$, and $T_{c_{\textit {ref}}}=\frac {|Grip~Force|}{Maximum~Grip~Force}$, and where ⌊·⌋ indicates the *floor* function, or rounding down to the next integer value. This command signal allowed the voltage sent to our tactor to range from 0.5-3.7 V for the corresponding range of $T_{c_{\textit {ref}}}$ values (0-1 corresponding to the value of *GripForce*). This voltage range resulted in supplying the maximum recommended current for the C2 tactor when the grip force produced was at its maximum, producing a vibration amplitude of 580 *μ*m. Conversely, a very small grip force produced noticeably weaker vibration amplitudes of 200 *μ*m and a null grip force produced no vibrotactile cue.

**Fig. 5:**
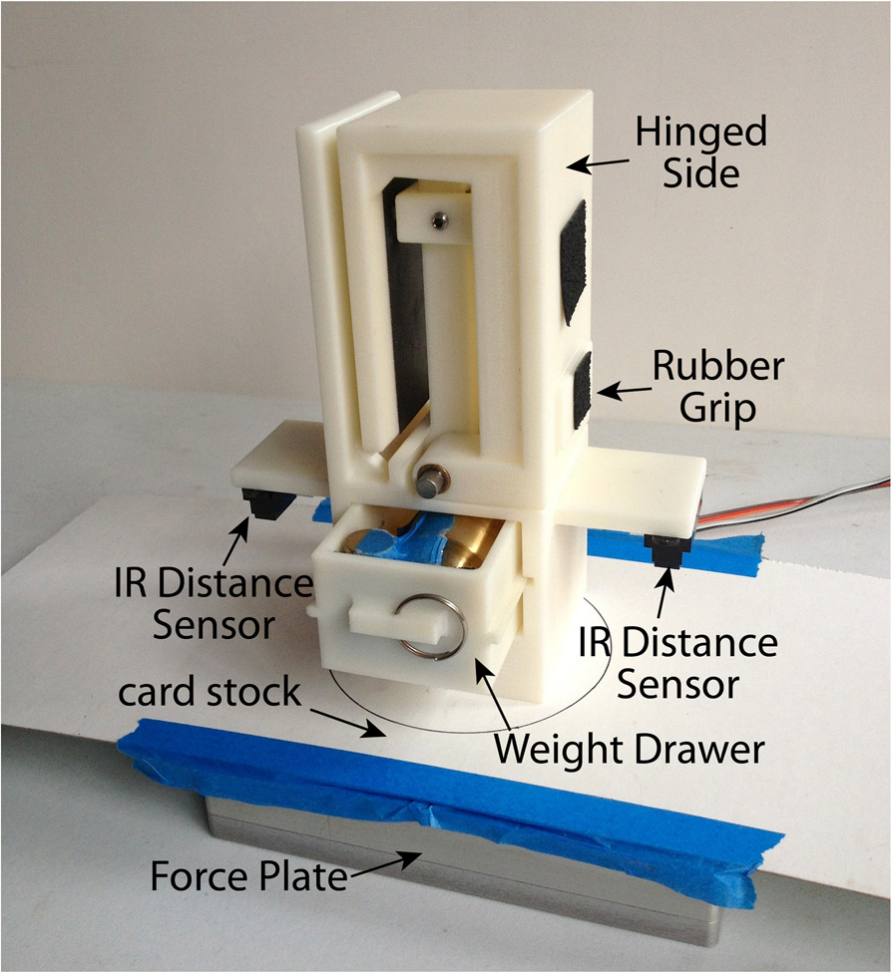
Instrumented testing object. Instrumented object with attached distance sensor and removable weight drawer atop a force plate. The card stock and force plate constitute the “table”

The instrumented object was a custom ABS plastic 3D-printed device with a removable drawer for inserting a weight. Rubber grips were placed on the side for grasping, and had a coefficient of friction: *μ*≃1 (determined experimentally). Two infrared distance sensors (Sharp 2D120X) were affixed to the object to measure vertical position. A 2 kg-capacity force plate (AMTI HE6X6-1) was used to measure the vertical load force. A piece of white card stock was attached to the top of the force plate to allow for more accurate position readings from the distance sensors (Fig. 5).

The entire system was controlled by a Sensoray 626 PCI card installed in a Dell OptiPlex 7010 series desktop running Microsoft Visual C++ 2010 Express Edition. The total weight of our gripper and exoskeleton was 2 kg.

### Experimental protocol

We tested N = 10 male participants, seven non-amputee (mean age 26.6) and three trans-radial amputees (mean age 53.3). Our non-amputee participants had no prior experience with myoelectric control of a prosthesis. All of our amputee participants were myoelectric users in the ‘experienced’ category who had used their devices everyday for at least six months. Prior to starting the study, each participant consented to a protocol approved by the Institutional Review Boards of the University of Michigan, Rice University, University of Houston, and Drexel University. Participants were not compensated, and testing lasted about two hours.

#### Training

Due to the design of the brace, all experimentation involved the right arm. The vibrotactile display was placed on top of the biceps area of the right arm and secured with the velcro sports band. The motorized brace was secured with velcro straps around the upper and lower portions of the right arm, aligning the elbow joint with the brace axis of rotation. The EMG gain on the conditioning circuit was adjusted to ensure the participant’s rectified and filtered EMG control signal was in the 0–5V range on an oscilloscope. The particitant was then instructed to maximally contract the muscles producing the EMG command *S*_*max*_, which was recorded by the computer. Then, the proportional control gain *K*_*g*_ and offset bias *G*_*b*_ were adjusted so that the participant could independently control the grip aperture and lifting motion. Although the gains and offsets were slightly different for each participant, the result was still the same: maximum gripper aperture when the EMG signal fell to zero, and minimum gripper aperture when the EMG signal was at its maximum. To ensure proper calibration, participants were required to successfully grasp and lift the instrumented object at the maximum weight three successive times. The joint torque feedback gain *K*_*e*_ was adjusted until it was independently recognized by the participant when grasping an object. The gain ranged from 1.0–3.5 depending on the participant. For vibrotactile feedback, $T_{c_{\textit {ref}}}$ was set based on the maximum grip force produced when the participant grasped an object.

#### Testing

The test consisted of 144 trials sectioned into four blocks of 36 trials each. There were three conditions being tested: vibrotactile feedback, joint torque feedback, and no feedback. The weight and condition were arranged based on a stratified randomization on two factors (object weight and feedback condition). Two weights were used, 340 g (drawer empty) and 590 g (250 g weight in drawer). During each block, each condition was presented 12 times and each weight was presented 18 times (note that a given weight was presented at least three consecutive times). Visual and auditory signals were not blocked during any of the trials so that participants could visually monitor task progression and hear all verbal instructions, including the audio chime (described below). Although amputees are known to use visual and auditory cues to estimate grip force, our instrumented object and gripper digits showed no visible deformation during grasping, nor did our gripper motor produce any of the distinguishable auditory cues common with geared transmissions.

A timer with bell chimes kept track of experiment progression. Each participant was given 10 sec to complete the task. This timing was used as a guide, and determined from pilot experiments. For each trial, the participant was instructed to start from a rest position, close and open the gripper, reach, grasp, and lift the instrumented object, then place it back on the force plate. Participants were instructed to grasp the object at the rubber grips and lift it a few inches off the force plate before returning it. After the 10 sec elapsed, or once the object was returned to the force plate if the participant took longer than 10 sec, the tester would remove the object from the force plate and change weights (per schedule) behind a cardboard curtain before replacing the object on the force plate for the next trial. This also took 10 sec (for the first participant, 15 sec were used). The participant was not aware of weight or condition changes prior to grasping and lifting the object. For every trial, an additional experimenter monitored whether a gross slip occurred while the object was in the grasp of the gripper, and hand-recorded the binary slip results. A break lasting a minimum of three minutes was taken after each block of 36 trials. Prior to starting each block, the control signals and feedback actuators were checked to ensure signal fidelity.

There were a few notable changes in the protocol for amputee participants. Amputees were not required to close the gripper at the beginning of each trial prior to grasping and lifting the object. This was done because the length of the motorized brace and mounted gripper often required amputees to rest the gripper against the table. Also, for amputee participants we only included the first two blocks of trials (1–72), based on results from the non-amputee participants. One amputee (participant 8) was given joint torque feedback in the form of a flexion moment rather than an extension moment because the participant could not feel the extension moment.

### Sample trials

**Fig. 6:**
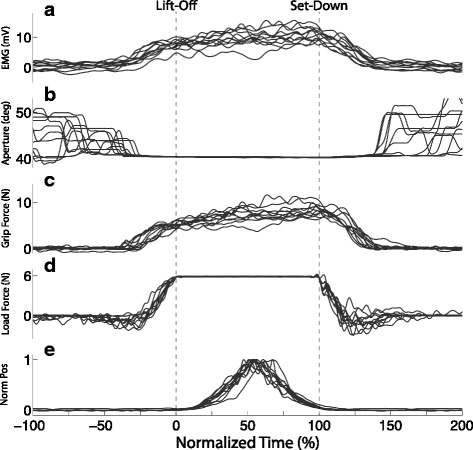
Time-domain plots for a representative non-amputee participant. **a** EMG, **b** Gripper Aperture, **c** Grip Force, **d** Load Force, **e** Normalized object position. All traces are for successful (no-slip) trials with the heavy object in blocks one and two (trials 1–72) with no feedback. Time has been scaled to object lift-off using a 99 % threshold on the maximum load force, and scaled to object set-down using a 98 % threshold on the maximum force for each trial

Figure 6 includes EMG, gripper aperture, grip force, load force, and normalized position traces for a representative non-amputee participant. For this particular participant, the 11 trials for the heavy object with no grip force feedback and no object slip are shown. There were no instructions on how high the object was to be lifted off the table. Time has therefore been scaled to object lift-off using a 99 % threshold on the maximum load force, and set-down using a 98 % threshold on the maximum force (to account for any contact artifacts) for each trial. Considering only the no-slip trials, the following was observed: gripper aperture followed EMG command until the gripper closed on the object (Fig. 6a,b); thereafter grip force tracked the EMG signal (Fig. 6a,c); grip force and load force increased prior to object lift-off and decreased to their initial state after object set-down (Fig. 6c,d); and object position traces were single peaked (Fig. 6e). These characteristics held across all participants.

### Metrics

To analyze task performance, assess the utility of haptic feedback, and compare non-amputee participants to amputee participants we examined the coordination between grip and load forces by means of time-domain and phase plots. Phase plots were generated by plotting grip force versus load force for all successful (no-slip) trials. To quantify the hysteresis in the phase plots, we computed the area inside the mean Grip/Load phase curves for the non-amputee and amputee participants, calling it *GripArea*. We also examined the grip force just before lift-off for all successful (no-slip) trials. (*GripT-10*) represents the grip force at T =−10 % on the scale determined by lift-off (t = 0 %) and set-down (t = 100 %). In addition, we examined the effect of object weight (heavy/light), participant type (non-amputee/amputee), and feedback condition (joint torque/vibrotactile/none) on the likelihood of an object to slip during a trial.

Due to complications during data collection, a few non-amputee participants did not complete all four blocks. However, all participants completed at least two blocks of the experiment. Four non-amputee participants completed all four blocks, and one completed three blocks. Therefore to stay consistent with our amputee participants, we will only focus on the first two blocks (trials 1–72) for our non-amputee participants.

### Statistical analysis

Linear mixed models (LMM) were used for the grip force at lift-off (GripT-10) and GripArea using SPSS (v.22) for estimating fixed and random coefficients. For each dependent measure, two sets of models were determined, the overall model and then distinct models for the non-amputee and amputee participant groups separately. Within the overall model, group, feedback condition, and object weight along with two-way interactions were fixed effects while participants were modeled as a random effect. The separate models for the non-amputee and amputee participant groups included feedback condition, object weight, and feedback condition × object weight interactions as fixed effects and participants as random effects. Bonferroni adjustments were applied to the estimated means to control for Type I errors.

The application of LMM allows for unequal numbers of observations per participant, does not require normality assumptions typically needed in parametric assessments, and is applied in repeated measures assessments [34]. The LMM model was fit by Maximum likelihood estimates (ML) and we tested the fit of the model using −2 log likehood chi-square statistic. For each measure, the simplest model with significant chi-square statistics indicated the best fit. After the models were fit, model assumptions were verified via visual inspection of residual plots. Visual inspection of residual plots did not reveal any obvious deviations from homoscedasticity or normality.

A Generalized Linear Mixed Model (GLiMM) was applied in development of a logistic model of the binary (yes, no), non-normally distributed slip data. The logistic model had a non-independence of measures along with the inclusion of random effects [35]. The probability of a slip occurring on a trial was modeled with participant group (non-amputee and amputee), condition (joint torque feedback, vibrotactile feedback, or no feedback), and object weight (light and heavy) as fixed effects, while participant was a random effect. A binary probability model was applied with the logit link function and the best covariance type was variance components [35]. The restricted expectation maximum likelihood (REML) was used for GLiMM model fitting. The Akaike Information Criterion (AICc), corrected for small sample size, was used for model selection. For each measure, the model with smaller values of the AICc indicated a better fit. Similar to the LMM, visual inspection of residual plots was done for verifying model assumptions. Our logistic model reports the omnibus results for object weight and participant group. The comparisons of feedback condition represent multiple comparisons. Indeed, these comparisons resulted in an inflated Type I error or multiple comparison problem. Using a Bonferroni correction for the overall multiple comparisons (5 - light vs heavy; amputee vs. non-amputee, none vs. joint torque feedback; none vs. vibrotactile feedback and vibrotactile feedback vs. joint torque feedback) results in a corrected alpha of *p*<0.01.

For all tests, a 0.05 significance criterion was applied.

## Results

Within minutes, our participants learned how to operate the proportional myoelectric low-impedance gripper using an EMG signal derived from the muscles in their forearm. Also, with little training they adapted their grip force for the weight of the object. Our participants were also proficient at coordinating their grip force with the load force as they lifted the object and set it back down, though differences did appear across the amputee and non-amputee groups as we describe below. We also examine differences in grip force coordination and the liklihood of the object slipping by participant group, object weight, and feedback condition.

### Grip force by weight

**Fig. 7:**
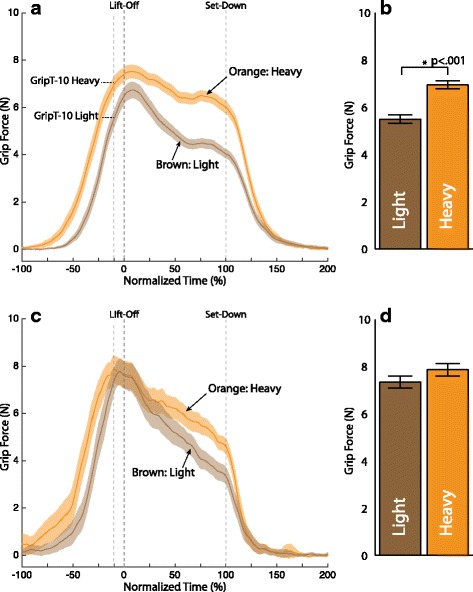
Grip force trajectory by object weight. **a** Non-amputee **c** Amputee participant. Solid lines represent the mean of all successful (no-slip) trials in the first two block (trials 1–72). Shading represents the 95 % confidence intervals of the mean. Brown traces represent the light object and orange traces represent the heavy object. The mean grip force for each weight just before lift-off *GripT-10* is shown in the accompanying bar plots:**b** non-amputee, **d** Amputee participants. Error bars represent 1 standard error

Although changes in object weight occurred throughout the blocks and were not made known to participants, we saw very little evidence of within-trial grip force readjustment on transition trials, as well as very little between-trial adaptation following transitions. Figure 7 shows the mean (with 95 % Confidence Interval (CI)) grip force trajectory for all successful (no-slip) trials in the first two blocks (trials 1–72). The mean grip force trajectories in Fig. 7 represent only the trials without weight transitions. The mean trajectories have been computed and plotted separately for each participant group (non-amputee (Fig. 7a) and amputee (Fig. 7c)). Trajectories are shown in overlay for the two object weights (heavy/light). Time has been scaled to object lift-off and set-down. For both participant groups, there are regions between lift-off and set-down where the 95 % CI bands do not overlap for the two weights. This indicates that participants on average used a larger grip force for the heavy object than for the light object. We can also see that the grip force increase begins earlier for the heavy object than for the light object. For non-amputee participants the grip force for the heavy object is greater while the object is just being lifted off the table. For amputee participants, the 95 % CI bands overlap in this region indicating that mean grip force was roughly the same for each object weight, though these 95 % CI bands seem to separate around time *t*=50 %.

Figure 7b shows a bar graph comparing the grip force just before lift-off (*GripT-10*) by object weight for the n = 7 non-amputee participants. Figure 7d compares the *GripT-10* values by object weight for the n = 3 amputee participants. The best fitting model (based on the likelihood test of the models *χ*^2^(1) = 10.435, *p*< 0.002) of *GripT-10* has fixed effects of intercept, group, object weight, feedback condition and group × object weight interaction with random effects of the intercept with subject as a covariate and an unstructured covariance. The significant fixed effects were intercept [F(1,11.2) = 73.34, *p* < 0.001], feedback condition [F(1,517.1) = 8.00, *p*< 0.005] and object weight [F(1,517.1) = 74.81, *p* < 0.001]. The variance of the random errors associated with *GripT-10* were randomly sampled from a normal distribution resulting in a variance of 2.98. For the non-amputee participants there was a significantly smaller grip force for the light object (*β*=−1.55,*SE*=0.16,*p*<.001) than the heavy object. There was no significant difference in *GripT-10* by weight for the amputee participants at lift-off.

### Grip force by condition

**Fig. 8:**
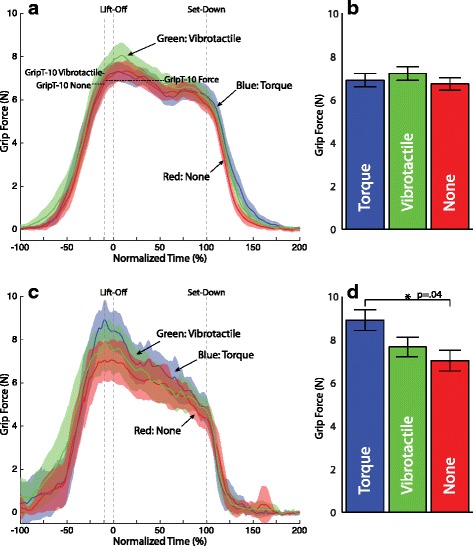
Grip force trajectory by feedback condition for the heavy object. **a** Non-amputee **c** Amputee participant. Solid lines represent the mean of all successful (no-slip) trials in the first two block (trials 1–72). Shading represents the 95 % confidence intervals of the mean. Blue traces represent joint torque feedback, green traces represent vibrotactile feedback, and red traces represent no feedback. The mean grip force for each condition with the heavy weight just before lift-off *GripT-10* is shown in the accompanying bar plots: **b** non-amputee, **d** Amputee participant. Error bars represent 1 standard error

**Fig. 9:**
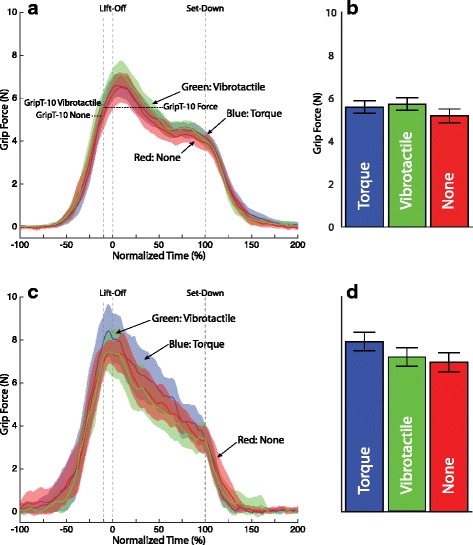
Grip force trajectory by feedback condition for the light object. **a** Non-amputee **c** Amputee participant. Solid lines represent the mean of all successful (no-slip) trials in the first two block (trials 1–72). Shading represents the 95 % confidence intervals of the mean. Blue traces represent joint torque feedback, green traces represent vibrotactile feedback, and red traces represent no feedback. The mean grip force for each condition with the heavy weight just before lift-off *GripT-10* is shown in the accompanying bar plots: **b** non-amputee, **d** Amputee participant. Error bars represent 1 standard error

Figure 8 shows the mean (with 95 % CI) grip force trajectory for all successful (no-slip) trials in the first two blocks (trials 1–72) for the heavy object only. The mean trajectories have been computed and plotted separately for each participant group (non-amputee (Fig. 8a) and amputee (Fig. 8c)). Trajectories are shown in overlay for each feedback condition (none, force, vibrotactile). Time has again been scaled to object lift-off and set-down. Overall, the 95 % CI bands overlap throughout the grasp and lift task, indicating that feedback condition made little difference on the grip force. However, there are a few time periods, particularly around lift-off where the 95 % CI band for one feedback condition is recognizably higher than the other two. For the non-amputee participants this is the vibrotactile condition. For the amputee participants this is the joint torque feedback condition. These same trends hold for the light object as well (see Fig. 9a and 9b).

The performance metric *GripT-10* was analyzed for the non-amputee and amputee participants for both the heavy object (Fig. 8b and 8d) and the light object (Fig. 9b and 9d) for each of the three feedback conditions. For the non-amputee participants there were no significant differences by feedback condition for either object weight. For amputee participants a significantly larger grip force was used during the joint torque feedback condition (*β*=1.32, SE =0.43, *p*=.003) than the no feedback condition for the heavy object. Their were no significant differences for the amputee participants for the light object.

### Phase plots

**Fig. 10:**
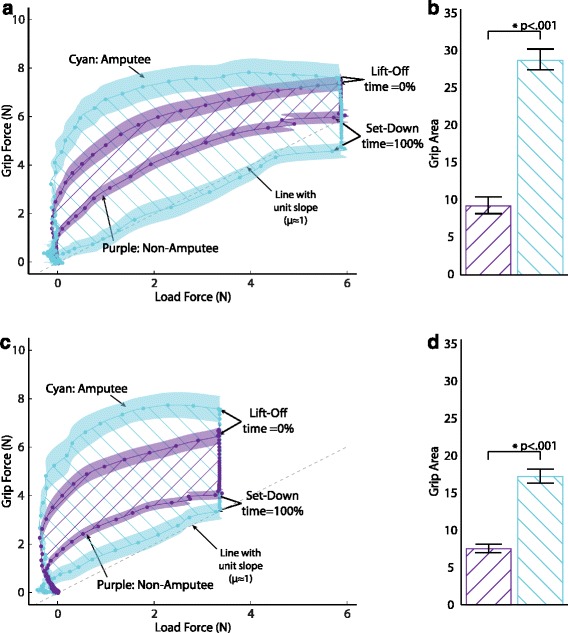
Grip load phase trajectory. Grip force vs. load force trajectory for non-amputee and amputee participants for the **a** heavy object and **c** light object. Dotted lines represent mean phase trajectory for all successful (no-slip) trials in the first two blocks (trials 1–72). Time has been scaled to object lift-off using a 99 % threshold on the maximum load force, and scaled to object set-down using a 98 % threshold on the maximum force for each trial. Each dot represents a 2.5 % *Δ*T of the normalized time vector. Shaded regions represent the 95 % confidence interval of the mean. Purple traces represent non-amputee participants. Cyan traces represent amputee participants. Dashed line represents the slip threshold. Hatch lines represent the area inside each trajectory (*GripArea*). The mean *GripArea* for non-amputee and amputee participants is shown in the accompanying bar plots for the **b** Heavy object and **d** light object. Error bars represent 1 standard error

To compare the coordination of grip and load force across participant groups, we generated the phase plots shown in Fig. 10. We plotted the mean grip force against the mean load force, computed separately for our 7 non-amputee participants and 3 amputee participants in all feedback conditions. Figures 10a and 10c show the grip versus load force for the heavy object and light object, respectively. Shaded regions around the mean traces represent the 95 % confidence intervals (CI). Dots along the traces indicate regular 2.5 % intervals of normalized time. The dashed line with unit slope represents the slip threshold (coefficient of friction *μ*≃1).

An increase in grip force generally preceded an increase in load force, but before the grip was fully developed, the load force ramped up in parallel with the grip force. While the objects were in the air (force plate unweighted to about 6N for the heavy object and 3.5N for the light object), participants relaxed their grip force somewhat. On set-down, participants relaxed their grip fully at the same time the weight of the object was taken up by the force plate. But differences are apparent in the grip/load force coordination patterns between our non-amputee and amputee participants. While lifting, our amputee participants used a higher grip force for the same load force than our non-amputees—a more conservative strategy for preventing slip. During set-down, however, our amputee participants used a lower grip force for the same load force than our non-amputees—a less conservative strategy. In short, the phase plot for amputees has more “hysteresis” than the phase plot for non-amputees.

The best fitting model (based on the likelihood test of the models *χ*^2^(1)=25.86,*p*<0.001) of *GripArea* has fixed effects of intercept, group, object weight, feedback condition and group × weight interaction with random effects of the intercept with subject as a covariate and an unstructured covariance. The significant fixed effects were intercept [F(1,32.7) = 5.929, *p*= 0.021], group [F(1,36.0) = 8.117, *p*= 0.007], object weight [F(1,498.6) = 4.827, *p*= 0.028] and group × object weight [F(1,498.4) = 29.387, *p*< 0.001]. The variance of the random errors associated with *GripArea* were sampled from a normal distribution with a variance of 73.89. The *GripArea* was significantly lower for the non-amputees relative to the amputee participants for the heavy (*β*=−19.44, SE =8.46, *p*=.05) and light (*β*=−9.57, SE = 3.77, *p*=.034) object weights, respectively.

### Likelihood of object slip

**Fig. 11:**
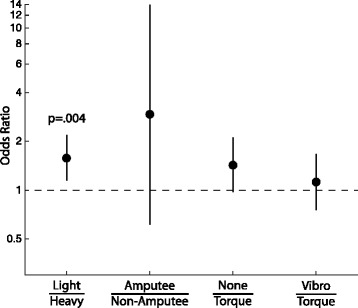
Odds ratio for object slip. Odds ratio are presented on a log-scale by conditions as follows: light compared to heavy object, amputees compared to non-amputees, no feedback compared to joint torque feedback, and vibrotactile feedback compared to joint torque feedback. An odds ratio of 1.0 represents an equal odds of slipping. Error bars represent the 95 % confidence interval

In order to develop the best possible model, we included slip data from all trials, including blocks 3 and 4 for those participants who completed them. Table 1 shows the number of times a gross slip of the object within the gripper was observed, broken down by object weight, participant group, and feedback condition. Pooling the weights, groups, or conditions selectively, we observed more slips for the light object than for the heavy, more slips for the no feedback condition than for joint torque feedback or vibrotactile feedback, and more slips for amputee participants than non-amputee participants. A logistic regression analysis for object slip found a greater likelihood of slip for the light object (*β*=0.46, 95 % CI[0.15,0.77], *p* =.004) than for the heavy object (Fig. 11). There were no significant effects for participant group although it is worth highlighting that amputees had a higher occurrence of slip than non-amputee participants. There were no significant effects for feedback condition. However, the greater likelihood of slip for no feedback (*β*=0.36, 95 % CI [−.02,0.74], *p*=0.064) compared to joint torque feedback is trending towards significance. Note that these results supersede our previous findings on object slip in [36].

**Table 1:** Slip descriptives (#slips/total)

Light object			
	None	Vibrotactile	Force
Non-Amputee	39/138 (28.3 %)	32/136 (23.5 %)	25/138 (18.1 %)
Amputee	18/39 (46.2 %)	15/41 (36.6 %)	14/40 (35.0 %)
Heavy object			
	None	Vibrotactile	Force
Non-Amputee	21/139 (15.1 %)	23/139 (16.5 %)	21/140 (15.0 %)
Amputee	16/42 (38.1 %)	12/41 (29.3 %)	16/40 (40.0 %)

## Discussion

In this study, we investigated how non-amputee and amputee participants performed a grasp and lift task using a backdrivable (low-impedance) prosthetic gripper whose aperture was controlled according to a surface EMG signal derived from a single muscle site in the forearm. Alongside this low-impedance mechanical design and novel control scheme, our prosthetic gripper conditionally referred sensed grip force to our participants through a vibrotactile actuator, or a motorized exoskeleton. In what follows, we discuss the influence of our backdrivable position-controlled prosthetic gripper on grasp and lift behavior, as well as the impact of referred haptic feedback of grip force on grasp and lift performance.

### Grip force was scaled to object weight

Our participants modulated their muscle activity to produce a grip force that was scaled to object weight (Fig. 7). Note that our participants could sense object weight (load force) through the forces and moments they supplied through the attachments between device and body to lift and balance the weight. Our seven non-amputee participants even produced a grip force before the weight of the object was fully known, as indicated by a grip force just before lift-off (*GripT-10*) that was significantly larger for the heavy object than for the light object (*p*<.001) (Fig. 7b). For our amputee participants, the difference in grip force did not appear until roughly halfway through the lift task.

The dependence of grip force on object weight in the early phases of a lift (before the weight is fully known) is indicative of a predictive or feedforward control strategy [31]. In our blocked randomized presentation of block weight, there was always a minimum of three consecutive trials with the same object weight. It is possible then that our non-amputee participants were employing predictive control schemes based on information from the previous trials. At the same time, we saw little evidence of within-trial grip force re-adjustment on transition trials, as well as very little between-trial adaptation following transitions. This is most likely the result of the large safety margins produced by our participants.

The evidence for feedforward action is less apparent for our three amputee participants, as differences in *GripT-10* were not significant. Feedback control, however, was used by both our non-amputee and amputee participants, as grip force was appropriately scaled for object weight by about half-way through the period during which the object was off the table.

It is worth considering here that the grip force traces produced by our participants uniquely differ from others found in the literature involving grasp and lift tasks with a prosthesis (see Figures 1 and 4 in [28] and Figures 3 and 5 in [14]). In particular, the traces produced by our participants show active modulation of grip force throughout the tasks, whereas the traces in the other studies show a plateauing behavior between lift-off and set-down. In [28], an open-loop rate-based control scheme was used in conjunction with a non-backdrivable (high-impedance) commercial gripper. In [14], a closed-loop position control scheme was used in conjunction with a non-backdrivable (high-impedance) research-based gripper. In both cases, the non-backdrivable nature of the devices necessitated the use of an antagonist pair of muscles to independently control device opening and closing. Therefore, it follows that any grip force produced as a result of an object in the grasp of the gripper would be maintained even after the muscle controlling device closure relaxed. In our setup, only one muscle controlled opening and closing, and therefore any grip force that was produced as a result of an object in the grasp of the gripper was only maintained while the muscle maintained its current level of activation. This is likened to the manner in which grip force is produced in our intact limbs (i.e. proportional to the current state of muscle activation).

The significant difference by weight in the grip force just before lift-off (*GripT-10*) for non-amputee participants and lack of significant difference for amputee participants might be explained given differences in the way participants from the two groups were fitted with the experimental apparatus. Since our non-amputee participants hand-held the gripper, they were privy, through receptors in their hand, to sensory information regarding object weight as transmitted through the gripper. For amputee participants, the weight had to be transmitted through the brace before it could be sensed. Also, the custom cuffs included a compliant connection between the rigid brace and each amputee’s residual limb that could have caused more uncertainty. The weight of our experimental apparatus (brace and gripper) was 2 kg, meaning the 250 g weight change, though recognizable, could have been masked by the apparatus, especially for our amputee participants. Certainly a viable prosthesis design would have to meet more stringent weight requirements.

### Marginal effect of haptic feedback on grip force

Our amputee participants increased rather than decreased their grip force when joint torque feedback was available for the heavy object in the no-slip trials. This strategy counters our expectations, but could be explained by considering that the chief aim of our participants was to prevent object slip. Providing grip force information in the form of joint torque feedback presumably allowed amputees to better monitor their grip force. Instead of using that information to control their grip force to a level just above the force needed to prevent slip as with grasp and lift for the natural hand, they appear to use this knowledge to ensure they were gripping with what they perceived to be a sufficient margin of safety while lifting the heavy object. This behavior is consistent with the manner in which it is anecdotally known that amputees use the auditory and visual estimate of grip force in their prescribed myoelectric prosthesis.

This strategy, however, was not carried over in the vibrotactile condition, suggesting that grip force may have been more difficult to monitor for amputees when provided as a vibration. Even still, the strategy of a larger grip force for the heavy object with joint torque feedback only worked 60 % of the time for the amputee participants (see Table 1). Yet, when we pool across object weights as well as the subject type (amputee or non-amputee), the object was 1.5 times less likely to slip with joint torque feedback than with no feedback, a result trending toward significance (see Fig. 11). It seems quite possible then that joint torque feedback has the potential to provide useful information regarding the amount of grip force being generated with a myoelectric prosthesis.

The lack of a significant effect of haptic feedback for our non-amputee participants is initially quite surprising given the effect of joint torque feedback for our amputee participants in the no-slip trials with a heavy object. This finding suggests, however, that our non-amputee participants utilized a feed-forward strategy, and relied on auxiliary haptic feedback, when needed, that was available to them through their hand rather than on the feedback provided through the vibrotactile or joint torque display devices. Our non-amputee participants held the gripper in their hand by a handle that likely transmitted cues regarding object weight better than the cuff worn by our amputees. In addition, contractions of the muscles in their forearm used for EMG control were accompanied by squeezing of the handle, with accompanying cutaneous cues and a sense of effort (from muscles acting about intact joints) that possibly supported the regulation of grip force and obviated the need for the vibrotactile or joint torque feedback provided.

Yet, haptic feedback has been demonstrated with non-amputee participants to improve the performance of tasks carried out using a prosthesis, including the grasp and lift task [6, 15, 28, 37]. Quite often, however, the utility of referred haptic sensation is often only apparent when feedforward uncertainty is present in the control loop [28], or when large safety margins on grip force have a negative effect on task performance, as is the case with brittle objects [15]. In addition, the utility of haptic feedback for non-amputee participants has only been demonstrated with high-impedance, non-backdrivable, prosthetic grippers. This is likely due to auxiliary haptic cues (in particular sense of effort) not always directly correlating with the grip force produced by these high-impedance grippers. When feedforward uncertainty became less reliable in these studies, haptic cues other than the auxiliary ones were needed to maintain performance. This suggests an apparent limitation of using non-amputee participants as a stand-in for amputee participants with a low-impedance backdrivable gripper. The muscles in the amputee’s residual limb no longer act about a joint, and it is possible that sense of effort is not available in the way it is for non-amputee participants.

Still, it is likely that the marginal effect of haptic feedback observed in this experiment is due in part to the highly dynamical experimental protocol. In each block of 36 trials, participants were presented with two object weights and three feedback conditions changing in a stratified random fashion without notice. In addition, participants were instructed to complete each grasp and list in a 10-sec window. This, combined with an instrumented object that was not brittle likely provided insufficient incentive to use the feedback to its utmost potential. Likewise, we did not perform any psychometric evaluations or in-depth training of our vibrotactile and joint torque displays to ensure participants understood completely how to interpret the information they provided. This latter point becomes more imperative when considering the many challenges associated with sensory substitution [38].

### A conservative grip/load force lift strategy, especially by amputees

As already noted, our participants coordinated their EMG command and lifting motions to produce a grip force that was roughly scaled to the load force. However, both our non-amputee and amputee participants produced grip/load phase plots that are non-linear and exhibit significant hysteresis. In particular, the phase plots demonstrate an overcompensating strategy (large safety margin in grip force) during lift. In contrast, there is a non-conservative strategy or even undercompensating strategy on set-down. The most economical scaling of grip force to load force, reflecting the unit-valued coefficient of friction for our gripper/object, would have produced a phase plot along the dashed line in Fig. 10. A phase plot along the load line determined by the friction coefficient is common in grasp and lift with the natural hand. See, for example, Figure 1B in [39], where a small safety factor is used to prevent the object from slipping.

Still, our participants do demonstrate a significant degree of coordination in that they began to lift the object before completing the ramp in grip force. The phase plots bend toward the unit slope line during lift. The existence of at least some simultaneous increase of grip and load force during lift-off is an indication of coordination, which is noteworthy given that our device differs so substantially from the natural hand. Coordination of grip and load forces is a unique characteristic of grasp and lift behaviors in the natural hand [1, 2, 31, 39–44].

The phase plots indicate that for a given load force, the grip force produced by non-amputees was smaller than that produced by the amputees during lift. During set down, however, the grip force produced by non-amputees was larger for a given load force. These differences in coordination of grasp and lift forces seen across our two participant groups is possibly due to the prior experience our amputees had using a myoelectric prosthesis that was not shared by the non-amputees.

Our amputee participants were each experienced using a commercial high-impedance, non-backdrivable myoelectric prosthesis in rate-control mode but were inexperienced with our particular control paradigm involving driving the aperture of a low-impedance, backdrivable gripper in proportion to an EMG signal derived from one muscle group in the forearm. This prior experience with very different device mechanics and control could have had a negative effect on the performance of our amputees, in that dexterous control would have required them to unlearn the control paradigm used for their current prosthesis before learning ours. The larger safety margin on lift-off could be a habitual behavior resulting from expectations appropriate only to their regular prosthesis. In addition, the under-compensation on set-down could arise from a tendency to want to use the atagonist muscle to open the gripper, and thus reduce grip force.

Our non-amputee participants had little to no exposure to myoelectric control, especially for grasp and lift tasks. However, our control paradigm did not prevent our participants from quickly learning to operate the gripper. Perhaps our selection of a muscle group for EMG control that is physiologically associated with grip in the natural hand was instrumental for the rapid learning.

We did not find significant differences in the amount of over- or under-compensation across feedback condition for either object weight, or either participant type. This is contrary to our original expectation that the amount of overcompensation would diminish when haptic feedback of grip force was provided. As mentioned previously, our experimental protocol may not have provided an incentive to use the feedback. It is possible, however, that this overcompensation had little to do with the availability of haptic feedback. In particular, it has been shown that even with a healthy intact hand, overcompensation of grip force can occur when the muscle commands are erroneously programmed for an object that is heavier than the actual object being lifted [39]. In our experiment, this confusion on object weight can be attributed more to a general level of uncertainty resulting from the stratified randomization of object weight and feedback condition, or our instruction to focus on preventing object slip. Indeed, our grip force traces (Figs. 7, 8, and 9) look very similar to those in Figure 2A in [39], as participants used a conservative grip force on lift-off, and then readjusted once the weight of the object became known (at T =−10 % and T =50 % for non-amputee and amputee participants, respectively).

### Object slip

The object slipped in least 19 % of the trials for our non-amputee participants (Table 1), suggesting that they were not always able to suitably modulate grip force with our position-controlled low-impedance gripper. This is likely due to our gripper denying our participants the full suite of sensory information that is available in the natural hand. This finding parallels the work of Augurelle et al. [22], who found that participants with anesthetized digits lack accurate coordination and make more mistakes in a grasp and lift task.

We saw significantly (*p*=.004) more slips for the light than the heavy object (Fig. 11), which suggests that grip force modulation was more difficult for the light object. Opening the gripper required a relaxation of the muscles generating the EMG signal, and closing the gripper required a contraction of those same muscles. Since neither of our participant groups had extensive training in modulating and maintaining EMG signals between these two extremes (maximum contraction vs maximum relaxation), it is possible that the desire to grip the lighter object with less force was met many times with the inability to maintain that lower force level.

Finally, when pooling participant groups and object weights, we found the object was almost 1.5 times more likely to slip when no feedback was provided than when joint torque feedback was provided. This result was not statistically significant, though it was trending toward significance.

## Conclusion and future work

In this study, we have only begun to explore the effect of both a position-controlled low-impedance gripper, and referred haptic feedback of grip force on myoelectric prosthesis performance. Using a grasp and lift task, we have observed that a position-controlled low-impedance (backdrivable) gripper driven by a single EMG electrode gives amputee and non-amputee participants the ability to coordinate and scale their grip force with the load force produced while lifting an object. This coordination and scaling is more consistent with behaviors observed using the natural hand (see Figures 1B and 2A in [39], noting that the set-down trajectory is omitted) than with high-impedance (non-backdrivable) grippers (see Figures 1 and 4 in [28] and Figures 3 and 5 in [14]). In addition, this coordination and scaling was more refined for our non-amputee participants than for our amputee participants, suggesting the influence of prior experience with the natural hand for our non-amputee participants, and experience with a commercial, high-impedance, rate-controlled prosthetic gripper for our amputee participants.

In terms of referred haptic feedback, our findings provide little conclusive evidence as to its overall impact and utility. Although we did see grip force regulation differences with joint torque feedback for our amputee participants, these differences were only present for the heavy object, and counter to expectations. In addition, these differences in grip force did not produce performance improvements in the form of reduced object slips. At the same time, however, the impact of joint-torque feedback was not replicated for vibrotactile feedback. It is possible then that grip force is more easily interpreted when displayed using joint torque feedback. As it stands, the lack of a psychometric evaluation in conjunction with our dynamic protocol does not allow for the study of these effects in isolation. Therefore, further testing needs to be conducted to validate this claim, taking into account the challenges associated with sensory substitution.

For our non-amputee participants, referred haptic feedback seemed to have no influence on grip force regulation or object slip. While this finding substantiates those from other studies involving high-impedance grippers, the use of a low-impedance gripper in this study suggests an alternative explanation. Namely, that non-amputee participants rely upon auxiliary haptic feedback (in particular, sense of effort) in combination with a feedforward strategy. This latter result alludes to a potential confound of using non-amputee participants as a stand-in for amputee participants when a position-controlled, low-impedance prosthetic gripper is used. As with our amputee participants, these conclusions need to be validated by further experiments that ideally use a less dynamic protocol.

We therefore envision additional studies to explore each of these findings in more detail. In particular, the utility provided by a low-impedance backdrivable prosthetic gripper under closed-loop position control should be weighed critically against the standard commercially available non-backdrivable prosthetic gripper, which offers reduced power consumption, weight, and user effort. Despite the benefits of fine force control akin to the natural hand, low-impedance back-drivable grippers tend to require larger actuators and constant activation of the muscles producing the myoelectric command. While the efficacy of low-impedance terminal devices has been demonstrated in body-powered prostheses and teleoperation more generally, the lack of significant findings for referred haptic feedback suggest that further research is needed for myoelectric prostheses. Still, the fact that participants in this study coordinated and modulated their grip force without prompting suggest that this form of force control may have come more naturally. This also suggests that haptic feedback may have a greater impact if joint torque feedback and vibrotactile feedback are compared in a less dynamic experimental protocol under the guidance of a psychometric analysis of each feedback modality. Beyond the work suggested here, however, the road to commercially viable backdrivable grippers will require the development of stronger and lighter actuators, better control algorithms, and improved mechanical design over the prototype presented in this study.

## Endnote

^1^ In the typical commercial myoelectric control scheme, the voltage across the motor terminals is driven in proportion to the EMG signal, such that when aperture motion is blocked by a hard grasped object, motor torque will be proportional to EMG signal. Thus proportional force control is achieved while grasping a hard object. When the aperture motion is not blocked, a voltage across the motor terminals in proportion to the EMG signal results in rate-control of the aperture.
